# Tetrahedral DNA nanostructures for effective treatment of cancer: advances and prospects

**DOI:** 10.1186/s12951-021-01164-0

**Published:** 2021-12-07

**Authors:** Jianqin Yan, Xiaohui Zhan, Zhuangzhuang Zhang, Keqi Chen, Maolong Wang, Yong Sun, Bin He, Yan Liang

**Affiliations:** 1 Department of Pharmaceutics, School of Pharmacy, Qingdao University, Qingdao, 266021 China; 2 National Engineering Research Center for Biomaterials, Sichuan University, Chengdu, 610064 China; 3 School of Biomedical Engineering, Sichuan University, Chengdu, 610064 China; 4 Department of Clinical Laboratory, Qingdao Special Servicemen Recuperation Centre of PLA Navy, Qingdao, 266021 China; 5 Department of Thoracic Surgery, Affiliated Hospital of Qingdao University, Qingdao, 266000 China

**Keywords:** Tetrahedral DNA nanostructures, Functionalized modification, Intellectualization, Drug delivery, Tumor treatment

## Abstract

Recently, DNA nanostructures with vast application potential in the field of biomedicine, especially in drug delivery. Among these, tetrahedral DNA nanostructures (TDN) have attracted interest worldwide due to their high stability, excellent biocompatibility, and simplicity of modification. TDN could be synthesized easily and reproducibly to serve as carriers for, chemotherapeutic drugs, nucleic acid drugs and imaging probes. Therefore, their applications include, but are not restricted to, drug delivery, molecular diagnostics, and biological imaging. In this review, we summarize the methods of functional modification and application of TDN in cancer treatment. Also, we discuss the pressing questions that should be targeted to increase the applicability of TDN in the future.

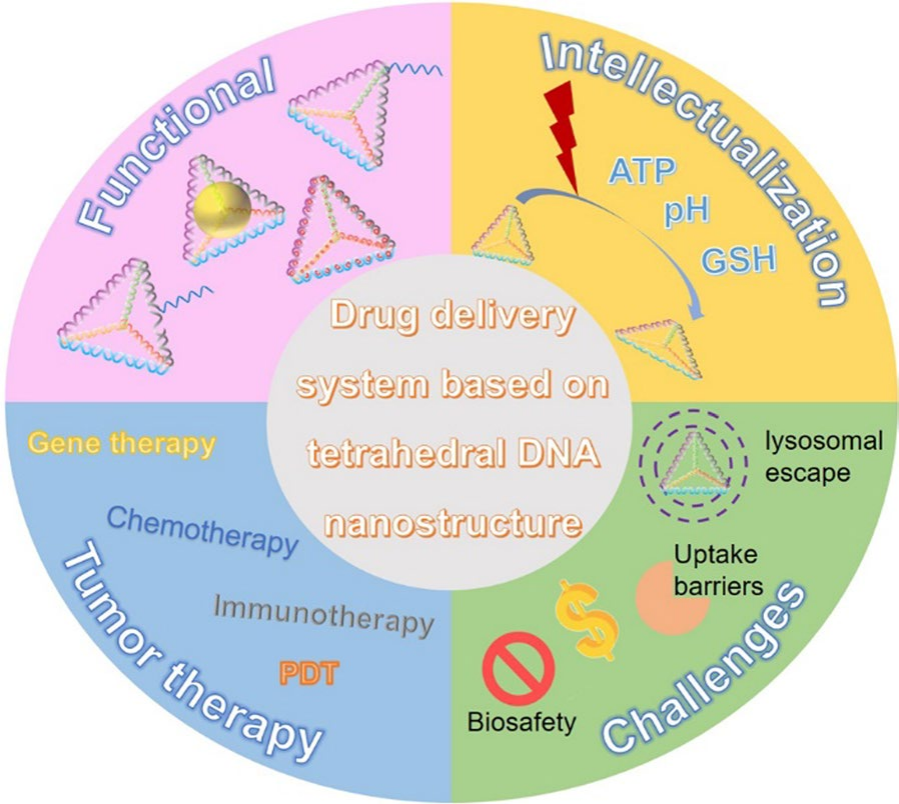

## Introduction

A safe and effective drug delivery system is urgently required to treat tumor growth, multidrug resistance, metastasis, and recurrence in cancer treatment. Multiple studies have been conducted to design and develop broad-spectrum of theranostic nanoplatforms to address this issue. The ideal nano-drug delivery system (NDDS) should simultaneously fulfill the following critical functions: (1) protect drugs from enzymatic degradation in vivo, (2) cross various physiological barriers, (3) provide accurate and controllable drug release, (4) reduce toxic and adverse effects of the delivered drug, (5) exhibit good biocompatibility and safety to the human body. Currently, multiple NDDSs are available, including organic nano-carriers such as liposomes [1, 2], polymeric micelles [3, 4], dendrimers [5, 6], metallic nanomaterial [7, 8] and inorganic nanoparticles like carbon nanotubes [9, 10], mesoporous silica [11], etc. [12, 13]. Although a variety of NDDSs have been used clinically, their heterogeneity, low biocompatibility, and low drug delivery efficiency limit the applications in cancer therapy. Therefore, it is important to increase the therapeutic index of drug delivery systems by developing innovative NDDSs with high clinical performance.

Deoxyribonucleic acid (DNA) is a biological macromolecule composed of four different deoxynucleotide monomers, which form supercoil structure by the complementary pairing of the basic group, thereby creating one the most vital biomolecules in the body [14]. The unique property of DNA molecules provides DNA nanomaterials unparalleled merits, including outstanding biocompatibility, good resistance to acidic and alkaline environment precise and adjustable structural control, and relatively straightforward computer-aided design of structure and function [14–17]. Compared with traditional NDDSs, the DNA nanotechnology is bringing revolutionary changes to the development of NDDSs for tumor treatment.

**Scheme 1:**
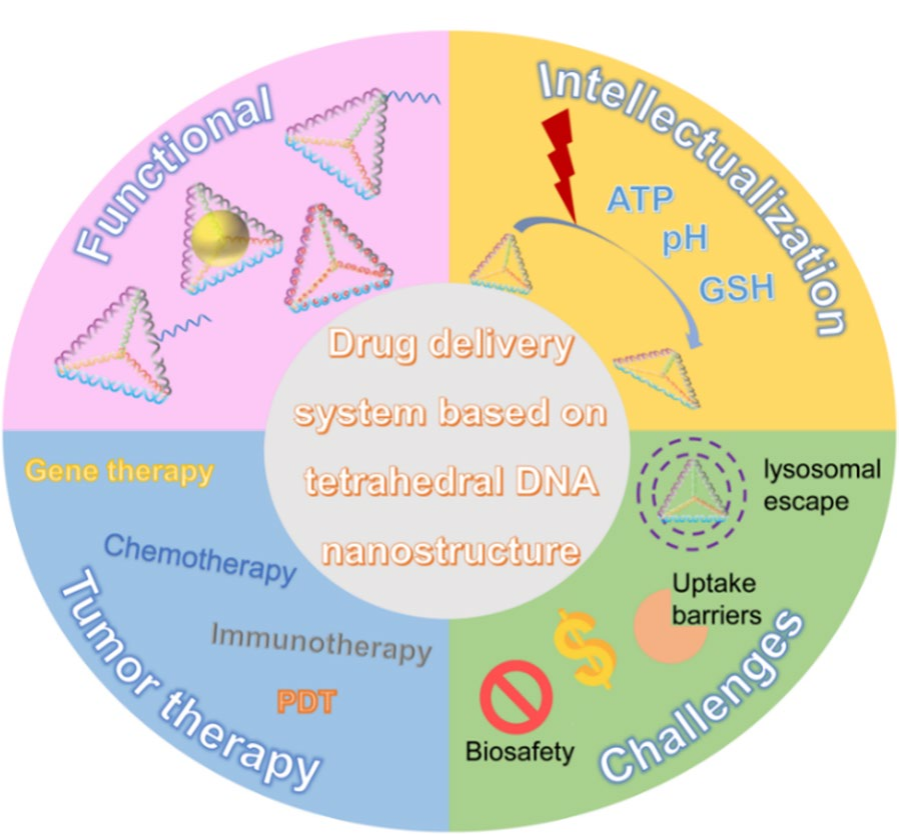
Design, application, and challenges of drug delivery system based on TDN

Tetrahedral DNA nanostructure (TDN) is a pyramidal three-dimensional nanostructure formed by the complementary pairing of four single-stranded DNA [14, 18]. TDN has been proposed as promising drug carriers due to their high stability, biocompatibility, rich functional modification sites, suitability for different drugs and excellent cellular uptake rates [19–22]. In Scheme 1, this review focuses on the functional modifications of TDN, intelligent NDDSs construction, and the prospects of TDN-based drug delivery systems for tumor treatment.

## Functional modification of TDN

**Fig. 1:**
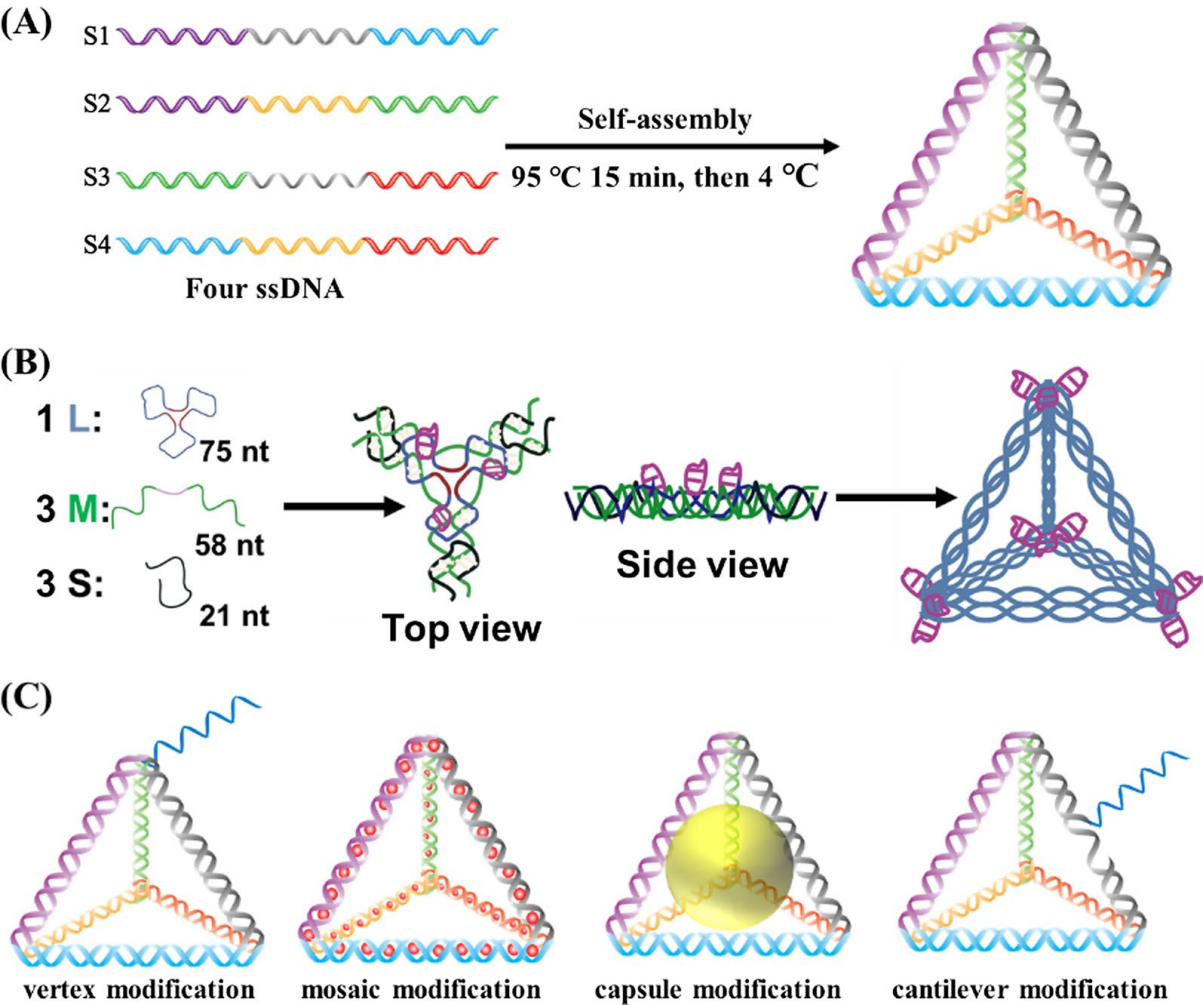
The schematic depicts TDN self-assembly (**A**) and double-bundle TDN [58] (**B**). **C** The key functional modifications of TDN are shown

Currently, TDNs are mainly used as duplexes and double bundles, among which the duplex TDNs are studied better. The functional modifications of TDN include fluorescent dyes [23–25], bioligand molecules [26], functional proteins [27], small molecule anticancer drugs [23], and even nucleic acid molecules [28], etc. (Table 1). According to the different positions of functional groups or molecules in the TDN, there are mainly four key modification ways, including vertex modification, mosaic modification, capsule modification and cantilever modification (Fig. 1).

**Table 1:** TDN modifications and their applications for drug delivery

Classification	Example	Modification	Application	Ref.
Small molecule	Doxorubicin (DOX)	Mosaic	Chemotherapy	[23, 29, 30]
	Paclitaxel (PTX)	Mosaic	Chemotherapy	[31]
	Platinum drugs	Mosaic	Chemotherapy	[32]
	Camptothecin	Cantilever	Chemotherapy	[33]
	5-Fluorouracil	Vertex	Chemotherapy	[34, 35]
	Methylene blue	Mosaic	Photodynamic therapy	[36]
	Triphenylphosphine	Vertex	Mitochondrial targeting	[37]
	Folate	Cantilever	Tumor targeting	[28]
	Actinomycin D	Mosaic	Antibacterial treatment	[38]
Proteins or peptide sequence	Cetuximab	Vertex	Immunotherapy	[39]
	Cytochrome c	Capsule	Apoptosis	[27]
	Streptavidin	Capsule Vertex	Immunotherapy	[40, 41]
	_D_-(KLAKLAK)_2_	Vertex	Mitochondrial targeting	[42]
	Angiopep-2	Cantilever	Receptor binding	[43]
	Tumor-penetrating peptide	Vertex	Tumor penetrating	[44]
	KillerRed	Cantilever	Photodynamic therapy	[45]
	Nuclear localization signal (NLS)	Vertex	Nuclear targeting	[46–48]
	Melittin	Capsule	Tumor treatment	[49]
Nucleic acid sequence	AS1411	Vertex	Tumor targeting	[50, 51]
	sgc8c	Vertex	Tumor targeting	[52]
	ZY11-targeting aptamer	Vertex	Tumor targeting	[53]
	17E DNAzyme	Vertex	Gene therapy	[53]
	siRNA	Cantilever Vertex	Gene therapy	[28, 30]
	Antisense oligonucleotides	Cantilever Vertex	Gene therapy	[45, 48, 54]
	CpG	Vertex	Immunotherapy	[15]
Other	Gold nanoparticles	Capsule Vertex	Tumor treatment	[55, 56]
	Gold nanoclusters	Vertex	Bacterial detection	[38]
	Anticancer metal complex	Mosaic	Tumor treatment	[57]

### Vertex modification

**Fig. 2:**
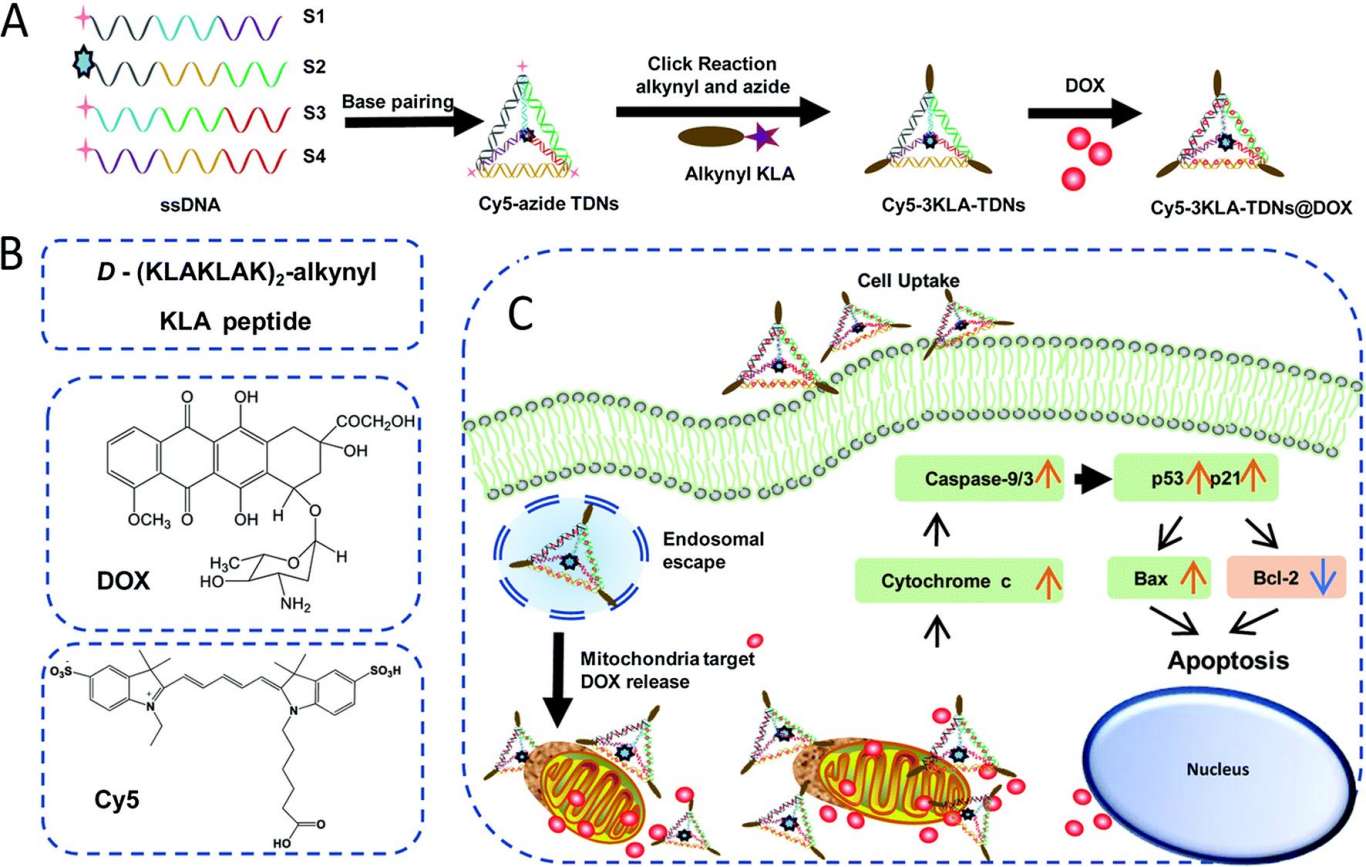
Schematic design of mitochondria-targeted 3KLA-TDN/DOX treatment for the breast cancer (Reprinted with permission from [42]. Copyright 2020, Royal Society of Chemistry)

Vertex modification refers to the modification of functional groups at the vertex position of a TDN, such as amino groups [59] or sulfhydryl groups [60, 61] used for TDN stabilization, specific sequence [59] or bioactive molecules used for molecular recognition, and azide groups [42] used for subsequent click reaction. In the process of vertex modification, functional groups are modified at the 5′- or 3′-end of SS-DNA, then the TDN is formed by self-assembly to make the 5′- or 3′-ends of four SS-DNA meet at the vertex of the tetrahedron. To enhance the therapeutic efficacy and targeting of breast cancer, Zhan et al. [34] attached the antimetabolite drug 5-fluorouracil (5-FU) to the TDN-based delivery system modified with a DNA aptamer (AS1411-T-5-FU). Anti-cancer reagent AS1411 could specifically bind to nucleolin, inhibit NF-κB signaling and reduce the expression of Bcl-2 [62–64]. Cell uptake research studies demonstrated that AS1411-T-5-FU has a better ability to target breast cancer cells than T-5-FU. At the same time, AS1411-T-5-FU and 5-FU were compared in terms of inhibiting cell proliferation and related protein expression. Mitochondrial apoptotic pathway evaluation showed that AS1411-T-5-FU could significantly upregulate the expression of Bax and caspase-3, down-regulate the expression of Bcl-2, and accelerate the process of apoptosis. Aptamer-based DNA materials have high recognition selectivity and specific binding to cancer cells, together with improving internalization efficiency. Yan et al. modified various numbers of _D_-(KLAKLAK)_2_ (KLA) to the apex of TDN and loaded the anticancer drug doxorubicin (DOX) to achieve the mitochondria targeting [42]. Related experimental results indicated that KLA-modified TDN could effectively deliver DOX to mitochondria and induce apoptosis. 3KLA-TDN exhibited improved cellular uptake, mitochondria targeting, apoptosis pathway activation and in vitro anticancer efficacy (Fig. 2).

### Mosaic modification

Mosaic modification means that functionalized molecules or groups are embedded in the double helix structure of TDN by conjugation, such as SYBR Green I [25] and other dyes [24] for fluorescent labelling, or anticancer drugs [23, 31–33, 42], and etc. DOX inhibits tumor growth by inserting DNA double strands to interfere with macromolecular biosynthesis [65]. DNA nanostructures loaded with DOX have the advantages of targeted delivery, response release, reduction of side effects and overcoming multidrug resistance, which are highly relevant for cancer and other diseases treatment. Dae-Ro Ahn’s group [23] prepared the DOX@Td (DOX loaded on the side of the DNA tetrahedral double helix by physical conjugation method) as a carrier for drug delivery analysis. By exploring the uptake mechanism of free DOX and the interaction of p-glycoprotein (P-gp) with cell membranes, it is found that DOX@Td entered cells through endocytosis and effectively overcomes multidrug resistance. Liu et al. [31] built a TDN drug delivery system loaded with PTX (PTX/TDN). PTX/TDN were efficiently transported into A549/T cells, avoiding drug efflux pumps because of the caveolin-dependent and exocytosis pathways. And PTX/TDN could significantly inhibit the proliferation of multidrug-resistant and wild-type cells. TDN may act as P-glycoprotein (P-gp) inhibitor, down-regulating the expression of mdr-1 gene and P-gp.

### Capsule modification

Capsule modification involves wrapping functionalized molecules in a caged structure inside TDN. Turberfield et al. [27] estimated that the central cavity of the tetrahedron can accommodate a sphere with a radius of about 2.6 nm. They bound cytochrome C to the 5′-end of oligonucleotide and changed the sequence of oligonucleotide to regulate the position (internal or external) of cytochrome C relative to the TDN. This design could be applied to initiate an apoptotic protease cascade. Mao et al. prepared the nanocomplexes with a class of core–shell structure by encapsulating gold nanoparticles in DNA cages [66]. Such complexes have promising application prospects in tumor treatment with photothermal, photodynamic and immunotherapeutic methods [67–69].

### Cantilever modification

Cantilever modification involves suspending functional molecules or groups on the side arms of TDN. For example, the intersection of the 5′ and 3′ ends of the SS-DNA is on the edge (middle or other non-vertex) of the TDN by designing the base sequence of SS-DNA, where the 5′ or 3′ ends without complementary pairing extend outwards for modification of functional molecules. Utilizing the hydrophilicity and editability of DNA nanostructures, Tian et al. [43] modified TDN with angiopep-2 (ANG-TDN), which showed a strong binding to the low-density lipoprotein receptor-related protein-1 (LRP-1) of glioma and the blood–brain barrier (BBB) cells. ANG-TDN was found to be stable in the serum for at least 12 h, indicating high stability. The modification of angiopep-2 could efficiently improve the uptake of TDN by brain capillary endothelial cells and Uppsala 87 malignant glioma (U87MG) cells. Meanwhile, experiments in vitro and in vivo showed that ANG-TDN could effectively cross the blood–brain barrier and precisely target U87MG human glioblastoma xenograft in nude mice. It had also been reported that siRNA or chemotherapy drugs can be loaded to TDN by cantilever modification [28, 33], and that exchanging the hydrogen bonds of branched DNA structures for covalent bonds can further enhance TDN stability.

Although different biomolecules had been linked to TDN for drug delivery, biological detection [59, 61, 70] and imaging applications [71, 72], it is still unclear whether TDN may carry molecules beyond its size and molecular weight. If different biomolecule is modified on the vertex or arm of TDN, the subtle balance of conformational flexibility of the TDN could probably be destroyed, resulting in the altered stability, rigidity, and geometric structures of obtained assemblies [73]. It is known that the size, shape, and number of charges of DNA nanostructures will affect their cellular uptake pathways, intracellular transport, and destination [74]. Whether attaching a nucleic acid with complex secondary structure will interfere with the TDN uptake process needs to be further explored [20], and choosing smaller sizes and charges is promising.

## Programmable TDN

The traditional NDDSs is generally not programmable, resulting in the drug being released once entering the organism, and the distribution in the organism is not selective, and eventually relatively large side effects. Only a few drug carriers could reach the tumor tissues through the enhanced permeability and retention (EPR) effect, but the drug has low bioavailability and poor efficacy. With the increasing understanding of tumor microenvironment, researchers have proposed building intelligent NDDSs to enhance antitumor efficacy. Because of the differences in the microenvironment of the tumor and normal tissues, the release of anticancer drugs at tumor sites can be controlled to improve their bioavailability and efficacy, meanwhile reduce their toxic and side effects on the non-affected organs. Programmability of TDN implies that the structure can recognise tumor microenvironment and target it. Programmable TDNs can recognize changes in pH [25, 75], excitation light wavelength [76], various components and their concentrations [77] to initiate modifications that meet different application requirements.

### pH sensitive TDN

**Fig. 3:**
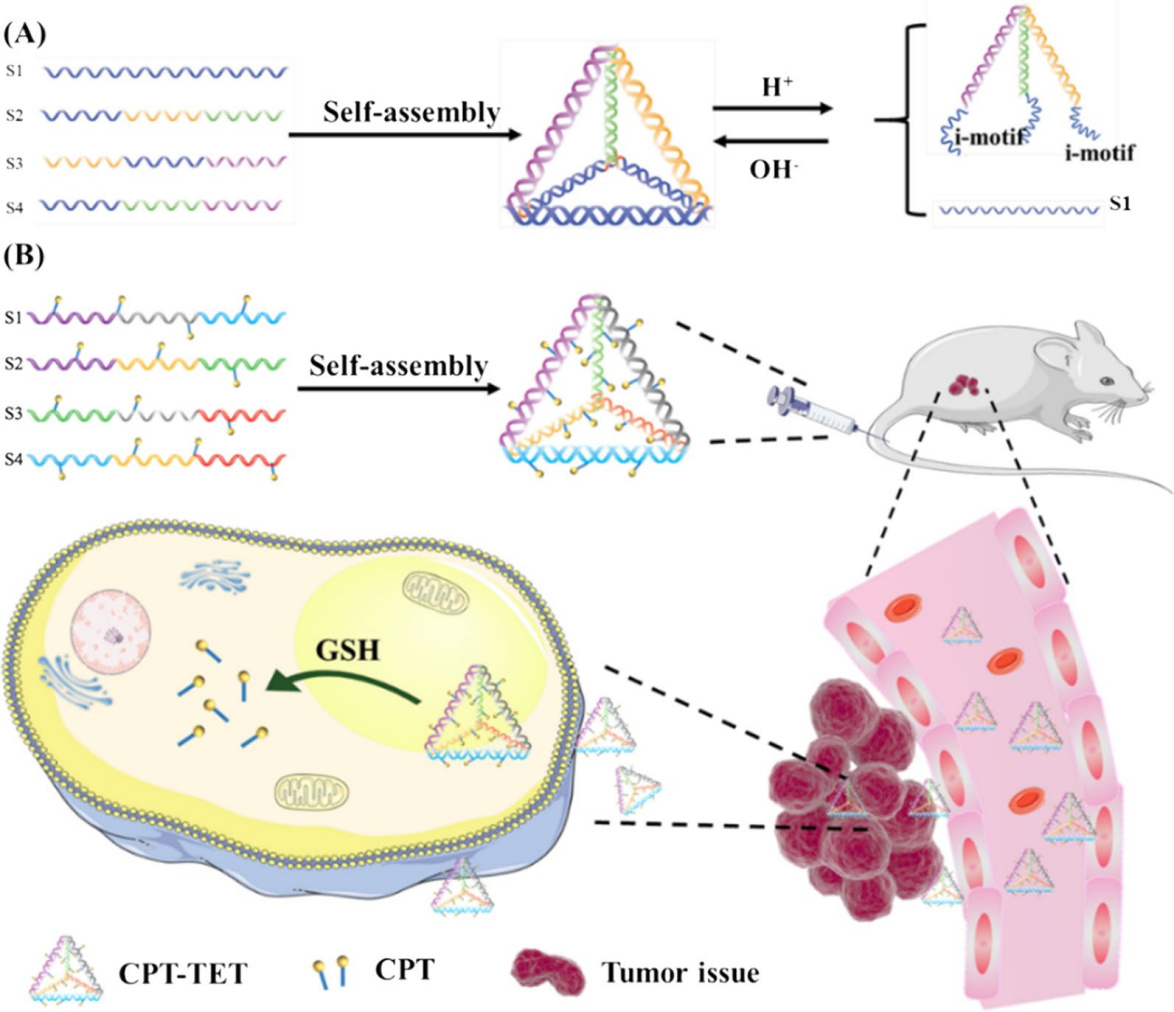
Programming the TDN. **A** Schematic shows pH-triggered conformational changes of TDN modified with an i-motif. **B** The synthetic route and delivery process of CPT-loaded TDN as precise and responsive nanomedicine

Wang et al. [78] monitored the changes of the TDN and the i-motif connected TDN at pH 8.5 and pH 4.5. The results showed that not only the orientation of the i-motif structure could be modulated electrically to produce an “open and close” signal, but the structure of TDN and DNA double helix would also change. Another experiment showed that the TDN structure loaded with DOX significantly increased the drug release under acidic conditions, which is related to the structural metamorphosis of DNA material [42, 44, 53]. Therefore, we speculate that the structure of the TDN deforms in an acidic buffer. At the same time, TDN can be purposefully modified to obtain pH sensitivity. Keum et al. [75] demonstrated a pH-dependent conformational change of DNA pyramids by introducing i-motif sequences (Fig. 3A). Their results demonstrated that the i-motifs can regulate the DNA pyramids assembly and disassembly and are suitable for in situ encapsulation and controlled release of proteins (enhanced green fluorescent protein, EGFP) by changing the physiologically relevant pH. Liu et al. [79] developed a strategy to reversibly assemble or disassemble DNA nanocages based on pH sensitivity. The pH-sensitive DNA tetrahedron was based on the DNA three-point star motif, which indicated that the DNA complex changed the structure between a single motif in a neutral solution (pH 8.0) and a tetrahedron in an acid solution (pH 5.0) to achieve the disassembly and disassembly of TDN. Kim et al. [80] proposed a method to encapsulate enzymes in TDN, which could change its conformation according to pH. TDN encapsulated the enzyme to avoid the degradation of the protein, reduce the binding of the enzyme and antibody, and reduce its activity. Due to the existence of i-motif-forming sequence and pH sensitivity, the conformation of TDN is changed, which promotes the enzyme to contact with other molecules. This approach can be further extended to reversible regulation of cell function through the pH-dependent activity control of enzymes. Such smart DNA nanostructure can potentially capture and release cargos on demand.

### GSH responsive TDN

Disulfide linkage, due to its stable and covalent linkages, have also been employed to control the DNA structure [81]. Endo et al. used disulfide linkage modified on the phosphorus atoms outside the DNA chain to connect two single DNA strands for branched DNA structures (XL-DNA) [82]. Two XL-DNA and complementary strands could self-assemble into multibranched DNA nanostructures. Glutathione (GSH) is an important reducing agent in cells and could efficiently cleave disulfide bonds. Therefore, multiple strategies were proposed for linking chemical compounds to DNA nanostructures with disulfide bonds. Zhang et al. [33] reacted phosphorothioate-modified DNA with carbonyl bromide-modified camptothecin (CPT) to form disulfide bonds (Fig. 3B). The DNA sequences grafted with CPT were then assembled into TDN structures using programmable DNA nanotechnology. The system could adjust the hydrophilicity of DNA-drug conjugates by regulating the amount and location of CPT modified on DNA to maintain its water solubility and molecular recognition ability. Programmable DNA nanotechnology could realize precise self-assembly of drug-containing TDN with stimulus–response properties and enhance antitumor efficacy in vivo and in vitro.

### Light responsive TDN

**Fig. 4:**
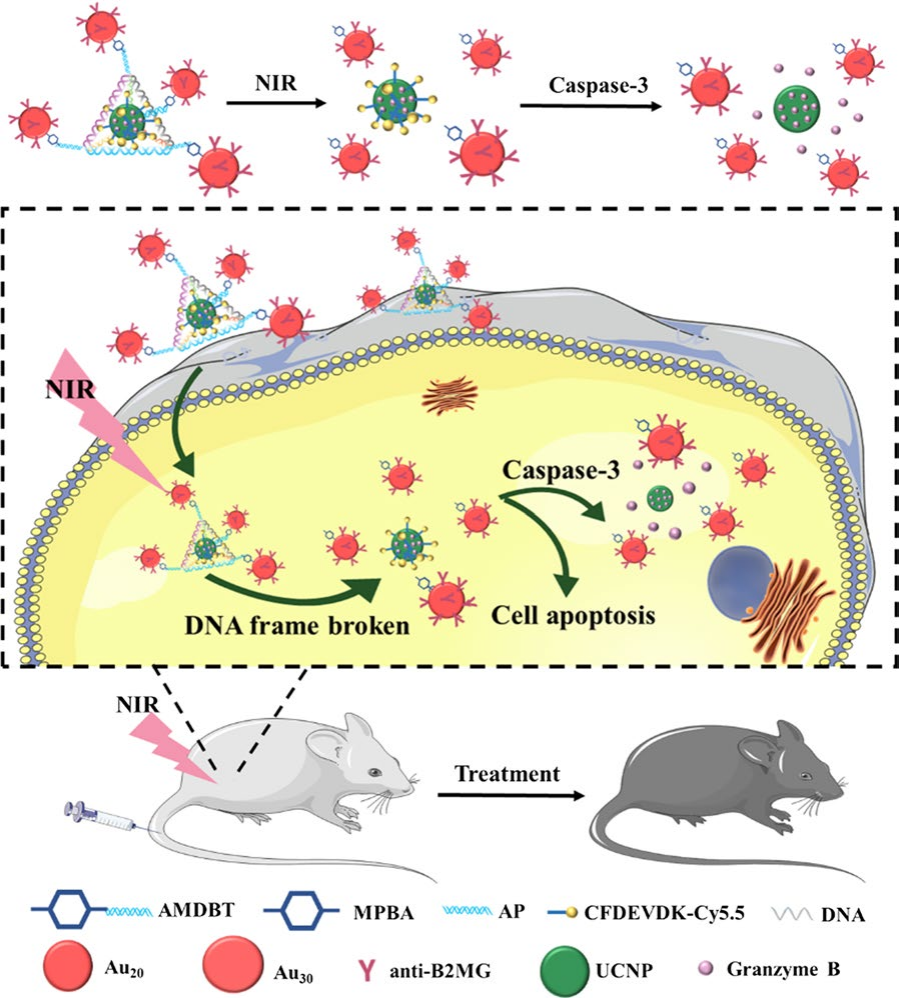
Schematic illustration of UAuTe tetrahedron used for senescence clearance

Han et al. [76] have successfully constructed a photon controlled TDN with azobenzenes. The shape of TDN can be controlled by alternating irradiation at different wavelengths. The results showed that the two isosceles of TDN were approximately 7 nm and the bottom edge was 11 nm before UV irradiation. After exposure to UV irradiation, some TDN contracted, causing the bottom side of the triangle to shrink to 4 nm. Triggering three-dimensional changes and promoting the release of cargos (such as proteins or other macromolecules) encapsulated in TDN allows for, precise temporal and spatial control. Quet al. [83] have successfully manufactured NIR-responsive upconversion-nanoparticle with Au20–Au30 centered in the nanoparticles tetrahedron (UAuTe) using DNA self-assembly (Fig. 4). The tetrahedron selectively targeted aging cells and induced the apoptosis of senescent cells by exposing Granzyme B under NIR light. When the β-2-microglobulin antibody (anti-B2MG) on Au NP recognizes senescent cells, applying near-infrared light (NIR) destroys the boronic ester linkage and induces the disassembly of UAuTe. In the presence of perforin, Granzyme B can induce target cell apoptosis via intrinsic adjustment. Compared with Granzyme B alone, the UAuTe could not only control the release of Granzyme B through NIR-responsivity, but also synergistically target the senescent cell and activate the Granzyme B without the need of perforin. The NIR-responsive TDN provides a practical strategy for aging and age-related diseases, and it also provides a potential for tumor therapy.

### ATP responsive TDN

**Fig. 5:**
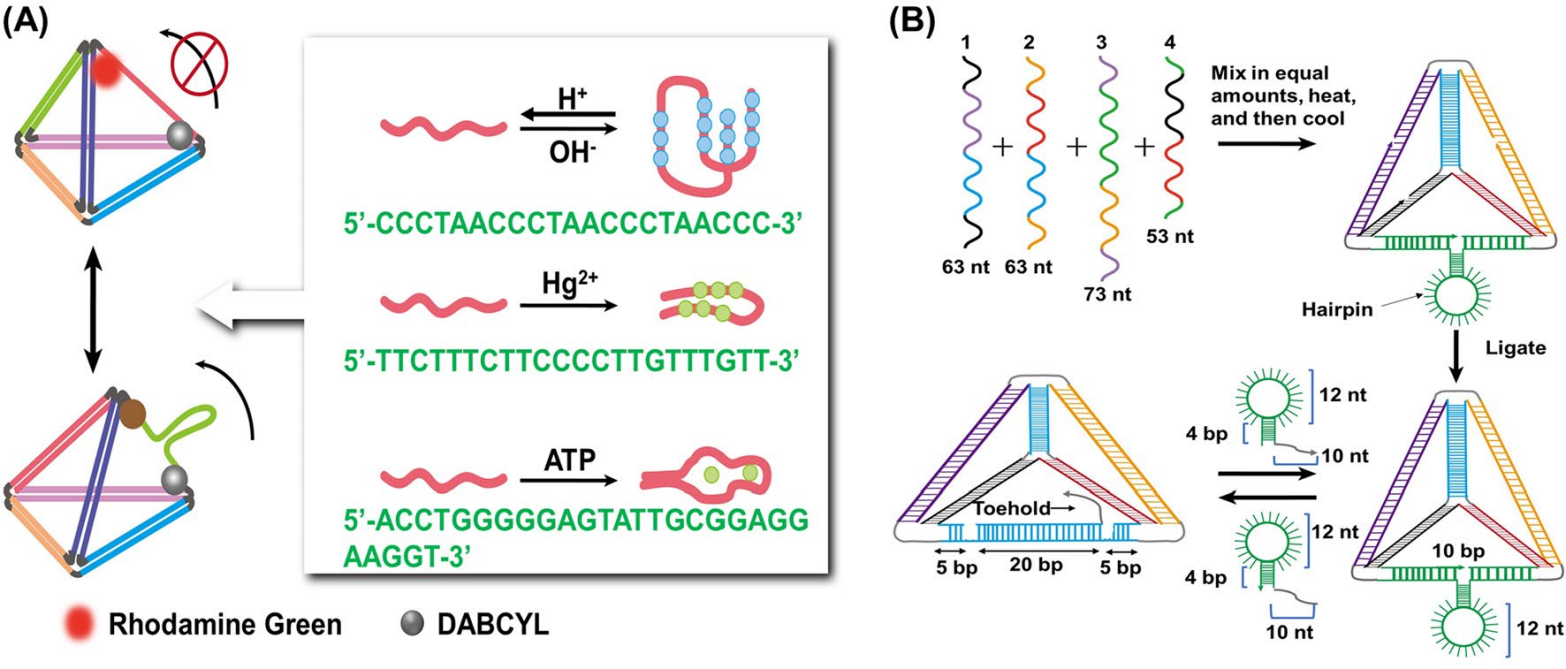
Programming the TDN. **A** The scheme demonstrates the TDN changes in response to the introduction of specific targets. Reprinted with permission from [77]. **B** Dynamic control of the TDN structure by specific sequences (Reprinted with permission from [89])

ATP-responsiveness is advantageous for the adjustment of DNA decomposition. ATP is the main energy molecule in the cells, and its concentration in the extracellular microenvironment (< 0.4 mM) is much lower than that in the intracellular microenvironment (1–10 mM). Moreover, the ATP concentration in tumour cells is higher than that in normal cells [84]. Based on these two differences, ATP-responsive DNA nanostructure DDSs can be designed [85]. Aptamer-ATP complexes are formed through conformational changes which promote the decomposition of DNA nanostructures and the release of drugs in ATP-rich environments [86]. ATP aptamers are commonly found in ATP-responsive DNA nanostructures because of their highly specific and sensitive properties [87]. Pei et al. [77] developed a DNA tetrahedron that showed a corresponding structural switching response to external stimuli (Fig. 5A). By adding dynamic sequences (i-motif, anti-ATP aptamer, T-rich mercury-specific oligonucleotide) to DNA tetrahedra, the configuration of the tetrahedron could be changed in response to the input of a specific target (protons, ATP, and mercury ions). These TDN provide new opportunities to “logically” control the release of drugs into cells.

### Dynamic control of the TDN structure

Abi et al. [88] showed that the reconfiguration switching of the tetrahedral structure could be efficiently realized under high ionic strength conditions. Goodman et al. [89] also studied and realised the dynamic control of a TDN structure designed with hairpin loops through specific nucleic acid sequences (Fig. 5B). In addition, the ability to reversibly switch the surface porosity of the nanocage helped to achieve the controllability of material transport in and out of the nanocage, which is a desired characteristic of a DDS. Zhang et al. [90] could reversibly switch the surface pore sizes of TDN by controlling two three-point-star motifs.

The response of TDN to the environmental stimulus to achieve different application requirements is the current research hotspot. Drug delivery to different types of cells requires different DDSs based on the physicochemical properties. These changes in conditions or components are stimulus factors and provide energy and impetus for structural changes. When these conditions are changed, the structure of TDN will change spontaneously and intelligently. Owing to the unique physical and chemical properties of DNA, the precisely modified sequences (i.e., i-motifs) can be embedded into the branched DNA endowed materials with different characteristics. The structure and size of DNA nanomaterials determine their cellular uptake pathways [74]. Whether these modifications affect their cellular uptake efficiency is still unknown. At the same time, the unbalanced stress distribution on the edges of the TDN corresponding to the DNA strings would result in the altered stiffness of the tetrahedron [20], and its mechanical properties and serum stability may change. These issues require further attention in future studies.

## Application of TDN in tumor therapy

### Chemotherapy

**Fig. 6:**
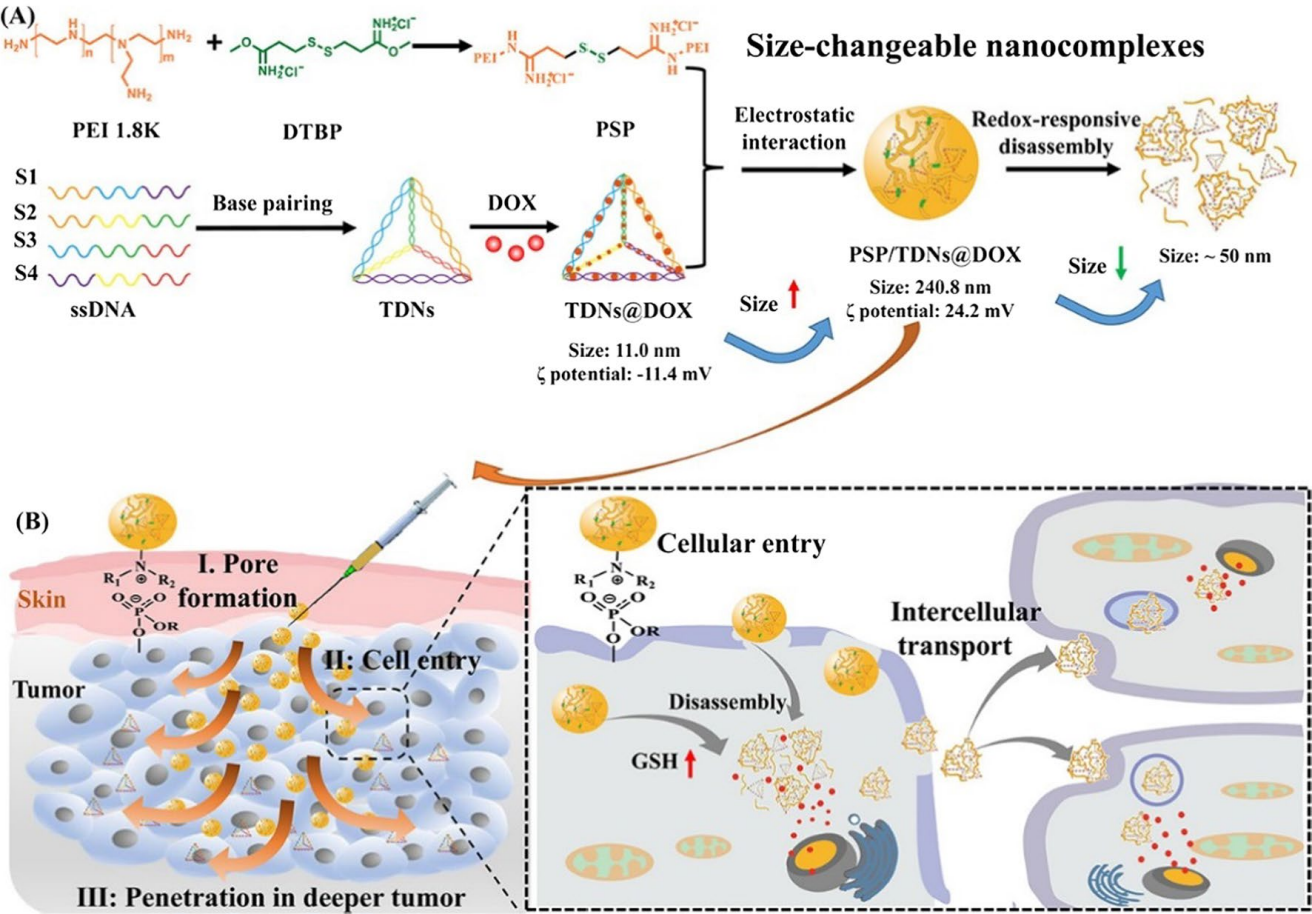
Schematic shows the strategy for modifying TDN with the redox-responsive polyethyleneimine. The presented functionalization method improves tumour cell/tissue penetration for treating multidrug-resistant tumours (Reprinted with permission from [96]. Copyright 2021, Elsevier)

Chemotherapy has a long history of clinical application and a wide range of indications, and it is currently one of the main methods for treating tumours. Most chemotherapeutic drugs are fat-soluble cellular drugs with different mechanisms of action, including: (1) affecting the chemical structure of DNA, such as cisplatin [91], (2) inhibition of nucleic acid synthesis, such as DOX and 5-fluorouracil [65, 92]; (3) interfering with DNA replication, such as camptothecin drugs [93]; and (4) interfering with the synthesis of tubulin during mitosis, such as paclitaxel [94]. At present, a variety of DDSs based on TDN have delivered the above-mentioned drugs to different organelles, such as mitochondria [37, 42] and nuclei [46], achieving excellent antitumour (even drug-resistant) effects both in vivo and in vitro. To solve the serum stability problem of natural _D_-sugar-based Td, Kim et al. prepared a mirror form of natural _D_-Td (_L_-Tds) and used it to load DOX [29]. The results showed that _L_-Tds could selectively deliver anticancer drugs to tumour cells and enhance cell/tissue penetration. At the same time, the mirror structure has an important effect on the pharmacokinetics and biodistribution of DNA nanostructures. In addition, we demonstrated that linking redox-responsive polyethyleneimine to TDN improve their serum stability by preventing enzymatic degradation, allow for tumor cell/tissue penetration, and overcome multidrug-resistant cancer [95] (Fig. 6).

**Fig. 7:**
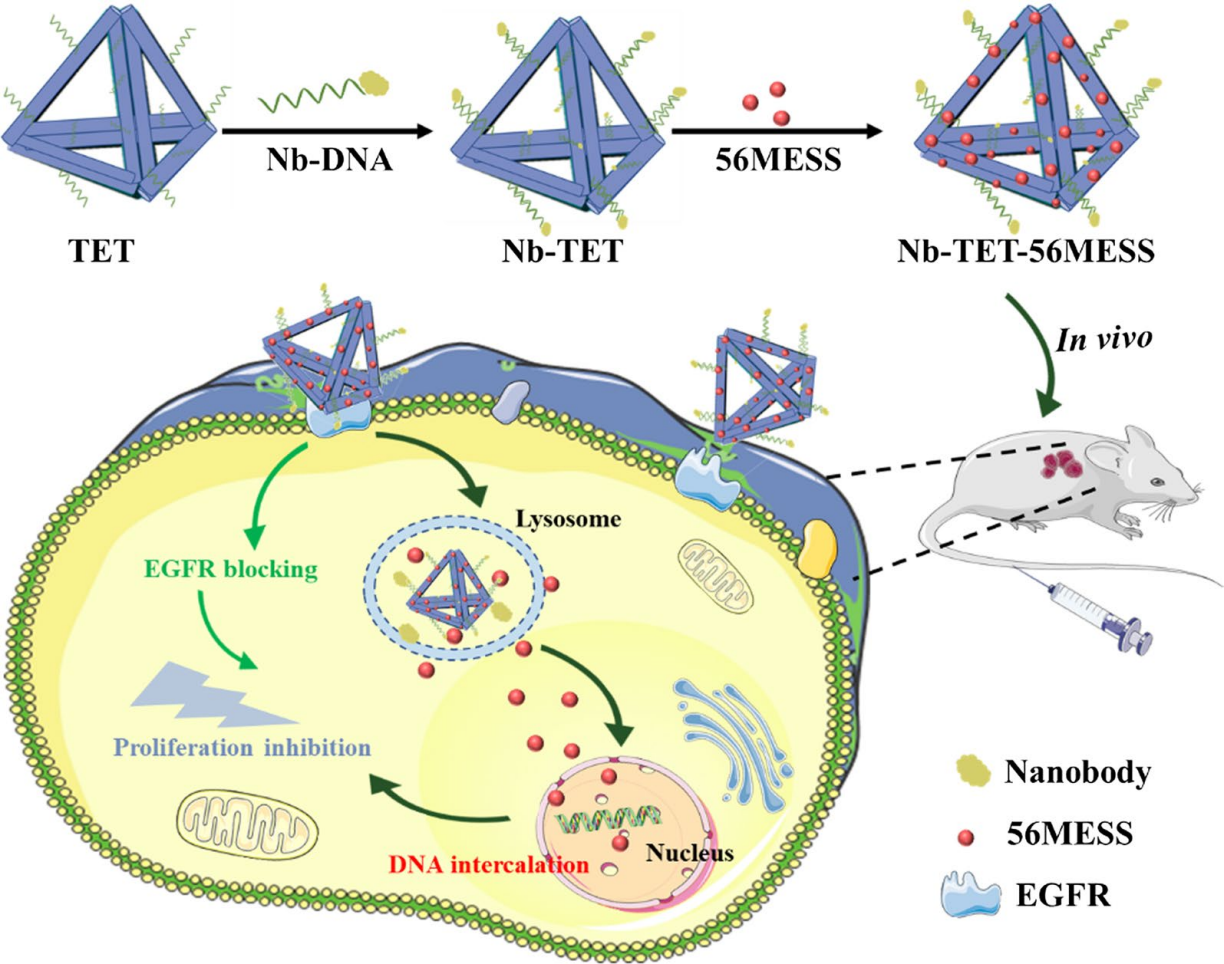
Schematic illustrates the nanobody-conjugated double-bundle TDN for targeted platinum drug delivery

Compared with duplex TDN, the double-bundle TDN invented by Mao et al. [58, 96] has better rigidity and stability, more modified binding sites and higher drug loading efficiency. Therefore, the double-bundle TDN has great potential as an efficient drug delivery system. Wu et al. [32] embedded the platinum drug 56MESS into a double-bundle TDN and coupled the anti-epidermal growth factor receptor (anti-EGFR) nanobody to the TDN to achieve multi-drug combination therapy for tumor (Fig. 7). The nanostructure could block EGFR signal transduction and exhibited excellent selectivity for cells with elevated EGFR expression, which exhibited significant anti-tumor activity without obvious systemic toxicity. In addition, drugs such as 5-fluorouracil, camptothecin and paclitaxel are loaded on TDN in different modified ways to achieve effective treatment of tumors [31, 33, 34]. The above-mentioned multifunctional DDS provides a new approach for tumor targeted chemotherapy and provides practical guidelines for enhancing reproducibility and reliability for the combined delivery of other functional components such as proteins.

### Photodynamic therapy

**Fig. 8:**
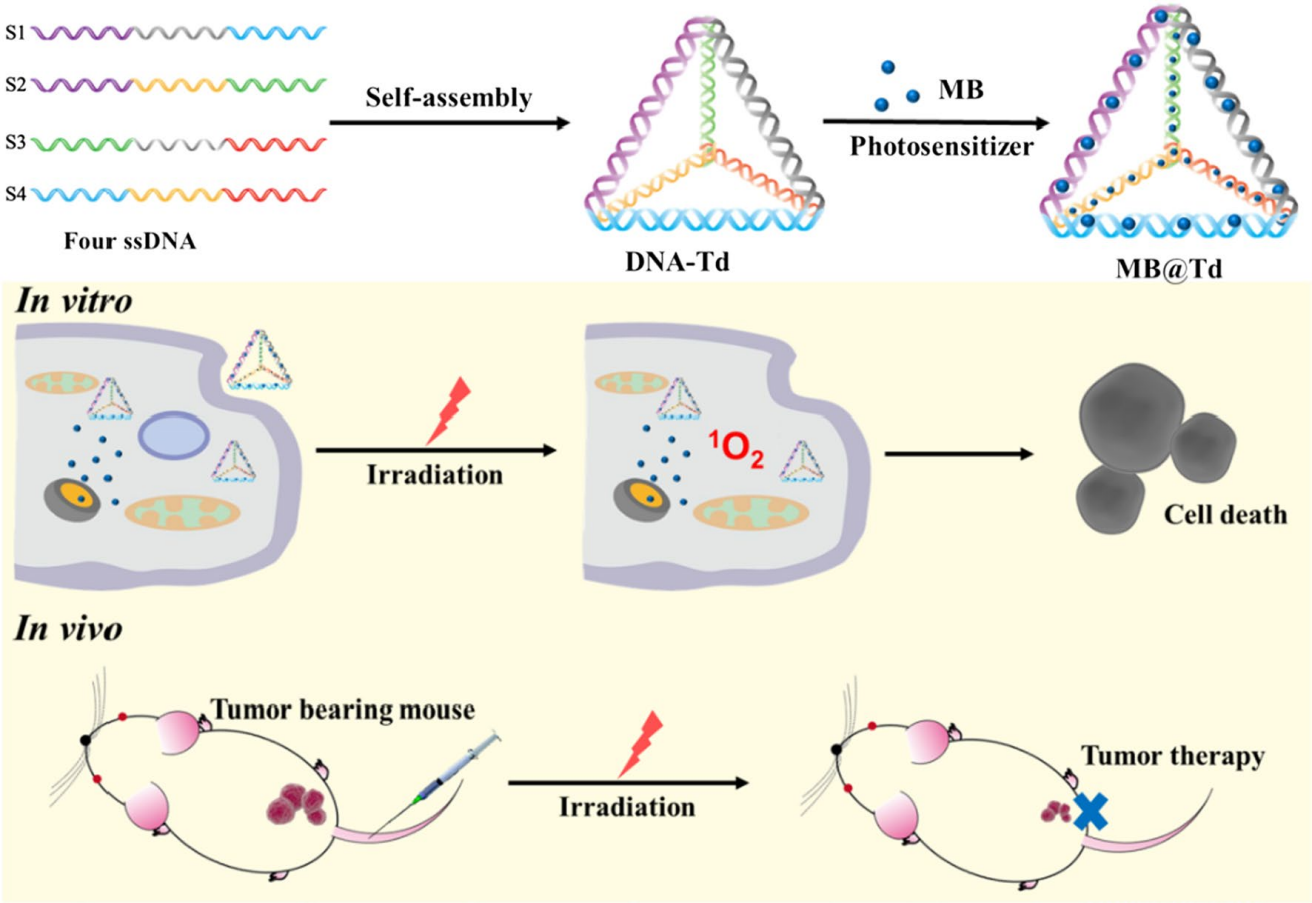
The schematic illustrates photodynamic therapy in vitro and in vivo using TDN loaded with methylene blue

Photodynamic therapy (PDT) is a minimally invasive therapy that has been clinically approved for cancer treatment with selective cell toxicity [97]. PDT has three main components: a light of specific wavelength, a photosensitising (PS) drug and oxygen. During the treatment process, the three interact to produce cytotoxic reactive oxygen species (ROS), which kill tumor cells through apoptosis or necrosis [98–100]. Typical photosensitizers, such as porphyrin derivatives and carbazole derivatives, have strong hydrophobicity and are easy to aggregate in aqueous solution, thus affecting the therapeutic effect [98, 101]. To this end, the researchers have developed various DNA nanostructures for the efficient delivery of photosensitizers. Kim et al. [36] employed TDN as a carrier for the intracellular delivery of methylene blue (MB) by taking advantage of the DNA binding property of the MB (MB@Td) and demonstrated photo-induced cytotoxicity (Fig. 8). Experimental results showed that sixteen molecules of MB could be loaded on TDN and delivered into cells without affecting the property of MB. The photo-induced cytotoxicity was virtually proportional to the amount of the intracellularly delivered MB in vitro. In addition, MB@Td produced an effective treatment effect of PDT and had a good tumor inhibition effect in vivo. TDN is expected to have superior properties for delivering PDT agents in future tumor therapies.

### Immunotherapy

**Fig. 9:**
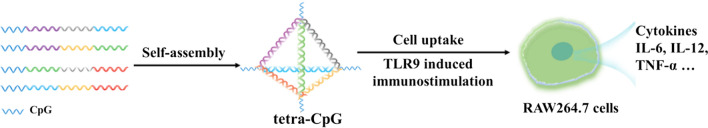
The schematic illustrates the assembly of CpG-bearing DNA tetrahedrons and their immunostimulatory effect

In recent years, tumor immunotherapy has attracted increasing attention as one of the most promising strategies for cancer treatment. In contrast to traditional radiotherapy and chemotherapy, immunotherapy use the host immune system to kill tumor cells and effectively inhibit tumor metastasis and recurrence. Cytosine-phosphate-guanine oligodeoxynucleotides (CpG ODN) can stimulate dendritic cells, B cells, macrophages to produce pro-inflammatory cytokines including tumor necrosis factor-α (TNF-α), interleukin-6 (IL-6), and activate the innate immune system by interacting with Toll-like receptor 9 (TLR9) to enhance anti-tumor activity [102–104]. The effectiveness of immunotherapy largely depends on the dose of the immunomodulatory sequences, and one of the critical problems is to increase the dose of agent in a single drug carrier. Functionalized materials can elicit specific immunological responses to therapy by incorporating special motifs with immunomodulatory activity. Therefore, CpG ODN, as a safe and effective vaccine adjuvant, has been widely used in basic research and clinical trials of tumor immunotherapy [105]. In recent years, the use of DNA nanostructures as CpG delivery vehicles has been explored. Owing to their inherent compatibility, CPG-rich sequences can be easily integrated into DNA nanostructures to enhance their stability and targeting. Liu et al. [40] used TDN to co-transport CpG and streptavidin (as a model antigen) to continuously induce a stronger immune response, and TDN alone did not elicit an immune response. Fan et al. [15] used TDN functionalized with unmethylated cytosine-phosphate-guanine (CpG) motifs for the immune activation of macrophage-like RAW264.7 cells (Fig. 9). The results of this study showed that the functionalized TDN are internalized by the cells and remain largely intact for 8 h, thereby inducing ample release of cytokines, including tumour necrosis factor (TNF-α), interleukin-6 (IL-6), and interleukin-12 (IL-12). Meanwhile, the multivalent CpG motifs also significantly enhanced the immunostimulatory effect of TDN. These studies have expanded the significantly of TDN in tumor immunotherapy and demonstrated the potential of further research in this direction. TDN can serve as a carrier for a variety of therapeutic agents and as stable vehicles for the target-delivery to immune cells or tumor cells.

### Gene therapy

With the continuous development of gene manipulation technologies such as gene editing and gene silencing, multiple approaches were proposed for up–downregulating the expression of target genes that are specific to the disease treatment. In recent years, gene therapy has gained increasing attention in the field of tumor treatment. A series of tumor-related genes, such as Ras, Myc and polo-like kinase 1 (PLK1), have been verified and used in clinical trials [106]. However, gene therapy drugs are not easily taken up by cells and are relatively unstable during circulation. Therefore, the success of gene therapy largely depends on the safety and effectiveness of gene delivery vehicles. Gene therapy vectors mainly include viral vectors and non-viral vectors. The use of viral vectors is limited because of possible insertion mutagenesis and immunogenicity [107]. Therefore, a major challenge for gene therapy is the design of non-viral vectors to achieve safe and efficient gene delivery. From the application point of view, TDN are advantageous as non-viral vectors owing to their inherent physiological effects, biocompatibility, and biodegradability. Due to the high loading capacity and high biocompatibility of DNA nanostructures, TDNs can be considered as non-viral vectors for effective targeted gene therapy.

Anderson’s group applied TDN nanomaterials to deliver small interfering RNAs (siRNAs) into nude mice model with tumors to inhibit the expression of target genes for tumor treatment research [28]. In this study, siRNA was suspended on the side of the tetrahedron by complementary pairing, and the tetrahedron loaded with the siRNA was targeted to the lesion site through the ligand connected to the cancer cell receptor. The hydrodynamic size of the nanoparticle is approximately 28.6 nm, which is favorable for cell uptake. DNA tetrahedron has a significant tumor-targeting ability after folic acid modification. Because the space direction of siRNA transported by TDN and the location and density of tumor-targeted ligands could be precisely controlled, the function of gene silencing could be maximized. Thus, tetrahedral DNA materials can be used for not only silencing the tumor target genes by delivering siRNA, but can also be used as a reference in the treatment of other diseases.

**Fig. 10:**
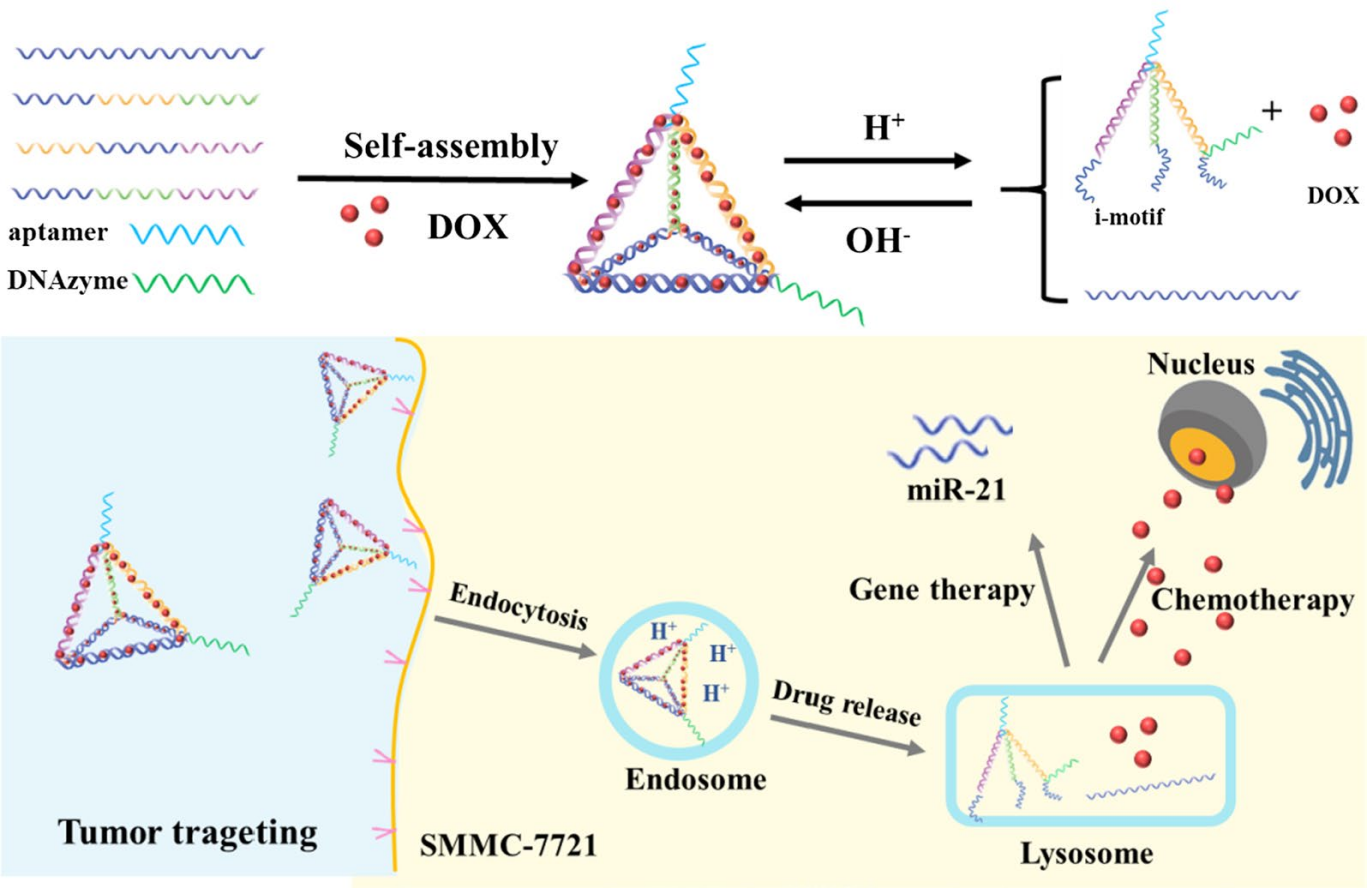
The schematic illustrates synergistic chemo-gene therapy targeting cancer cells

Despite the emerging evidence demonstrating exciting achievements, there is still much room for further development of TDN. As single treatment no longer meets the high demands for the efficiency of tumor treatment, the combined application of multiple treatments become a promising research field. Zhong et al. [53] reported a nanocarrier consisting of TDN, ZY11-targeting aptamer, DOX and 17EDNAzyme to achieve synergistic chemo-gene cancer therapy (Fig. 10). In addition, the combined use of DOX and CpG could achieve chemo-immune combination therapy. Therefore, the applicability of traditional treatment methods can be further expanded by developing new strategies for tumor growth inhibition.

## Challenges and prospects

TDN has decisive advantages that make them promising novel drug carriers. TDN can be synthesized easily, reproducibly, and has good biocompatibility and excellent performance. Through reasonable modification, TDN can efficiently incorporate chemotherapeutic drugs, nucleic acid drugs, imaging probes and etc., and exhibit good application potentials in drug delivery, molecular diagnostics, and biological imaging. However, TDN still have weaker sides such as poor enzyme resistance and insufficient ability to cross physiological barriers, including the lack of targeting, poor permeability in tumor tissues, and low cell entry efficiency. For efficient drug delivery, several roadblocks have to be removed before TDN can compete with existing drug carriers such as polymers, liposomes, and inorganic nanoparticles.

First, adverse factors such as enzymatic degradation and protein adsorption in the body can destroy the structural integrity of TDN, causing the untimely drug leakage and failure to reach the expected target site. He et al. and Lin et al. showed that packaging with polyethyleneimine (PEI) or PEGylated protamine could significantly avoid TDN enzymatic hydrolysis, promote the cellular uptake and lysosomal escape of TDN [95, 108, 109]. Lin et al. also also demonstrated that multiple pathways, including micropinocytosis and caveolin- and clathrin-dependent endocytosis contributed to the endocytosis of PEI/TDN complexes. However, the toxicity or other adverse effects of these chemical modifications still need to be considered. For example, PEI 25 K has greater cytotoxicity [110], therefore, it is urgent to explore better solutions.

Second, the lysosomal escape ability of TDN needs to be further improved. Although DNA nanostructures could enter cells better than oligonucleotide, Fan’s group found that TDN was trapped in lysosomes after entering cells [46], prompting the collapse of TDN and the release of drugs. Some antitumor drugs, whose targets are not in the lysosome (pH value about 4.5–5.5) [111] and are unstable under acidic conditions and release in lysosomes could make them less effective or inactivated. Their group modified the nuclear localization signal peptide (NLS) at the TDN vertex to synthesize NLS–TDN and endowed it with nuclear targeting capability, which helped the TDN escape from lysosomes and accumulated in the nucleus. Therefore, improving the lysosomal escape ability of TDN is particularly important for the maximising therapeutic effect of drugs. Furthermore, to expand the structural and functional diversity, a more refined design of the structure and introduction more kinds of stimulating components (such as metal-sensitive, and magnetic-sensitive components) are needed.

Third, there are still barriers that hinder the effective transportation of DNA nanostructures including TDN in the process of drug delivery, and the barriers include the blood–brain barrier and plasma membrane barrier. Fan et al. [46] confirmed that the entry of TDN into cells in a caveolin-dependent endocytosis pathway through a series of fluorescence imaging and biochemical experiments, which is a type of receptor-mediated endocytic pathway. However, these energy-dependent or receptor-recognized pathways cannot efficiently deliver DNA nanostructures, such as TDN. Therefore, it is particularly important to design TDN-based drug carriers that can effectively penetrate various physiological barriers, specifically target diseased tissues or cells, with little or no uptake by normal organs and cells [112].

Biosafety is another concern. Oligonucleotides are biodegradable and biocompatible. However, things may change when DNA is designed into nanostructures. The dynamic unstable state and thermodynamic stable state of nanostructures should conduct more in-depth research on the physical and chemical properties of TDN. The potential immunostimulatory properties of TDN must be systematically investigated before they can be used for clinical biological applications [113]. Meanwhile, research on the pharmacokinetics of TDN (in vivo circulation, distribution, metabolism, etc.), the effects on liver and kidney systems, and whether it will cause harmful genetic recombination are not sufficiently deep [114, 115]. A few studies have suggested that TDN is mainly excreted by the kidney [116–118], which may be detrimental to the accumulation of TDN in tumors. The biosafety of TDN and other DNA nanostructures will be the focus of future research. We believe that these explorations in immunostimulatory properties and pharmacokinetics as well as the actual conformation of branched DNA will provide better guidance for tumor suppression and immune surveillance in a more predictable manner.

Finally, at present, in vivo experiments related to TDN are still mainly conducted in mice, and there are still many challenges before conducting related experiments in humans. The main problem is the cost of production. For practical biomedical applications, high-purity functional DNA nanostructures must be produced in sufficient quantities. Several groups have reported convenient and cost-effective purification methods for DNA nanostructures at the laboratory level, but these methods have not been demonstrated on a larger scale. At present, the purification methods that have been reported for DNA nanostructures include agarose-gel-based separation method [119] and ultracentrifugation [120], and methods such as asymmetric PCR, RCA and fermentation are used to control costs and produce DNA in large quantities [120]. However, this remains a far cry from cheaper polymers. Improving the purity of DNA nanostructure, especially TDN, and reduce the cost is a practical problem that needs to be considered in the application.

## Conclusions

Efficient drug carriers based on DNA nanostructures represent a promising goal of future research. Further increasing the yield of DNA nanostructures, exploring the mechanisms of cellular entry, overcoming biological barriers to improve cellular internalization, and controlling production costs are the major challenges. With the development of new strategies and technologies, including molecular design, assembly, and applications, we envision that DNA nanostructures will gain broader applicability as intelligent drug delivery carriers in the future.

## Data Availability

Not applicable.

## Abbreviations

TDN: Tetrahedral DNA nanostructures
NDDSs: Nano-drug delivery systems
DNA: Deoxyribonucleic acid
5-FU: 5-Fluorouracil
DOX: Doxorubicin
KLA: _D_-(KLAKLAK)_2_
P-gp: P-glycoprotein
BBB: Blood–brain barrier
EPR: Enhanced permeability and retention
EGFP: Enhanced green fluorescent protein
GSH: Glutathione
CPT: Camptothecin
anti-B2MG: β-2-Microglobulin
NIR: Near-infrared light
anti-EGFR: Anti-epidermal growth factor receptor
PDT: Photodynamic therapy
PS: Photosensitizer
ROS: Reactive oxygen species
MB: Methylene blue
CpG ODN: Cytosine-phosphate-guanine oligodeoxynucleotides
TNF-α: Tumor necrosis factor-α
IL-6: Interleukin-6
TLR9: Toll-like receptor 9
CpG: Cytosine-phosphate-guanine
PLK1: Polo-like kinase 1
SiRNAs: Small interfering RNAs
PEI: Polyethyleneimine
NLS: Nuclear localization signal peptide
